# Pediatric vascular anomalies in Austria. Where are we at? A survey among primary care pediatricians

**DOI:** 10.3389/fped.2023.1216460

**Published:** 2023-08-17

**Authors:** Paolo Gasparella, Simone O. Senica, Georg Singer, Chiara Banfi, Christina Flucher, Besiana P. Beqo, Holger Till, Emir Q. Haxhija

**Affiliations:** ^1^ Department of Pediatric and Adolescent Surgery, VASCERN VASCA European Reference Centre, Medical University of Graz, Graz, Austria; ^2^ Faculty of Medicine, Riga Stradins University, Riga, Latvia; ^3^ Institute for Medical Informatics, Statistics and Documentation, Medical University of Graz, Graz, Austria; ^4^ Boston Children’s Hospital and Harvard Medical School, Boston, MA, United States

**Keywords:** vascular anomalies, health organization, rare conditions, patient journey, infantile hemangioma

## Abstract

**Introduction:**

Vascular anomalies (VAs) are rare conditions and affected patients often experience a difficult patient journey. Timely diagnosis is only possible if primary caregivers are aware of the anomalies and are connected with dedicated specialists. Aim of our survey was to investigate the knowledge about diagnostic and therapeutic possibilities for children with VAs, and the existing networking among primary pediatric caregivers in Austria.

**Methods:**

Primary care pediatricians in Austria were invited to complete an online questionnaire consisting of 28 questions focusing on pediatric VAs.

**Results:**

Out of 373 invited pediatricians 93 (25%) returned the questionnaires, 86 of which were complete. Most physicians (39/93 42%) answered that they see between 15 and 30 patients with infantile hemangiomas per year. Vascular malformations are rarely treated in the primary care setting; most primary care pediatricians (58/86, 67%) reported that they currently treat fewer than 5 patients with such type of VAs. There was unequivocal agreement among the participants (84/86, 98%) on the need to establish a network of specialists and a registry dedicated to pediatric VAs.

**Conclusions:**

This survey represents the first study shedding light on the awareness of VAs among Austrian pediatricians and can serve as a basis for future investigations and advances in the management of these conditions in Austria and other countries with a similar healthcare setting.

## Introduction

Vascular anomalies (VAs) represent a variety of mostly rare conditions affecting any body part and involving each type of vessel (1). Their incidence varies according to the type of VA and it is high for small infantile hemangiomas (up to 1 out of 10 newborns), while very low for vascular malformations and syndromes (up to 1 out of 5,000 newborns) (2). VAs may present at birth or manifest later during the pediatric or even the adult age. Unfortunately, since most VAs are rare conditions, diagnostic misclassifications or delays can considerably postpone the initiation of the optimal treatment (3, 4). According to a recent study based on medical records from the UK and US, the time needed for a correct diagnosis of any rare diseases may take up to 7.6 years (5).

The International Society for the study of Vascular Anomalies (ISSVA) introduced a classification for VAs in 1996. Based on the clinical and histologic behavior of vascular lesions, tumors and malformations were separated according to the presence of endothelial proliferation. Tumors are further subdivided depending on their aggressivity while malformations differ according to the type of involved vessel.

Dedicated centers with expertise in VAs facilitate both a correct diagnosis and an appropriate treatment. However, primary care pediatricians (PCPs) play a fundamental role in referring patients and their families to these centers (6). Furthermore, their role is also vital to inform the patients' families about facilities such as patients' associations providing additional psychological and organizational support.

Unfortunately, the lack of awareness of the need for a timely diagnosis of VAs and the scarce networking with dedicated specialists increase the risk of late and incorrect diagnoses (7). Education and information could help increase the awareness of rare diseases and trigger clinical suspicion to generate more accurate diagnoses of VAs (8).

The aim of our study was therefore to investigate the knowledge of PCPs about the diagnostic and therapeutic processes, as well as the medical network of VAs in Austria.

## Materials and methods

Between January and February 2021, Austrian PCPs were invited by email to complete an electronic survey focusing on VAs. Twenty-eight questions were developed aimed at investigating five specific topics: (1) characteristics and profile of expertise of the PCP; (2) number of patients with VAs treated by the PCP; (3) knowledge of current protocols for the treatment of VAs; (4) networking with centers of reference for VAs or patient associations; (5) opinion on future development. The complete questionnaire is shown in Table 1.

**Table 1:** Survey items with their response options.

1	In which region do you work as pediatrician? Burgenland Carinthia Lower Austria Upper Austria Salzburg Styria Tyrol Vorarlberg Vienna
2	Distance between your outpatient clinic and the closest regional hospital <30 min 30–59 min 60–89 min 90–119 min More than 120 min
3	How many patients aged 18 or less do you treat at the moment? <250 250–499 500–749 750–999 1,000–1,249 1,250 or more
4	Which of the following departments have you worked in? Pediatric hemato-oncology Pediatric emergency room Pediatric surgery Pediatric dermatology
5	How many cases of infantile hemangioma do you see each year? <15 15–30 >30
6	In which percentage of such cases did you start treatment? <5% 5%–10% 11%–15% >15%
7	Do you have experience with therapeutic options for the treatment of childhood hemangiomas? Yes No
8	Which of the following therapeutic options for **infantile hemangiomas (IHs)** do you have experience with? Topical beta-blockers Systemic beta-blockers Surgical removal (excision) Laser therapy Systemic corticosteroids Others (specify)
9	In case treatment is necessary, you treat only isolated small lesions, otherwise you send the patient to a reference center you send the patient to a reference center you treat the patient yourself
10	Which of the following parameters are decisive for IH evaluation and treatment? Size Localization Number
11	Minimum age for IH treatment initiation: At the latest in the 2nd month of life At the latest in the 4th month of life At the latest in the 6th month of life After the 6th month of life
12	How many patients with VMs do you treat at the moment in your outpatient clinic? <5 5–10 11–20 >20
13	How many of these patients are currently treated with anticoagulants or compressive bandage? none 1–5 6–10 >10
14	How many of these patients are currently treated with surgical or radiological techniques? none 1–5 6–10
15	Which of the following statements is true regarding your treatment and diagnosis of these pathologies? I consult a specialist who has experience in this field I consult an interdisciplinary group that deals with these problems I refer to a reference center only for complicated cases I am not aware of any centers or specialists that deal with these pathologies
16	Do you have patients with chronic vascular malformations who need support from a patient association? Yes No
17	Do you know any patient association? Yes No Which ones do you know? (specify)
18	Do you recommend a particular patient association? Yes No Which one do you recommend? (specify)
19	Do you know the ISSVA classification? Yes No
20	Which of the two statements do you agree with? The ISSVA classification is essential for the correct treatment of a vascular anomaly Although I am aware of the ISSVA classification, I do not consider it essential for the correct treatment of vascular anomalies.
21	Do you know the AIVA working group? Yes No
22	I have participated in the annual meeting of the working group AIVA Yes No
23	I know publications of the AIVA working group Yes No
24	Do you think that the national educational opportunities are sufficient to address the problems associated with vascular anomalies in depth? Yes No
25	I would like to learn more about this topic Yes No
26	I find it important to focus on more common pathologies Yes No
27	It is important to establish a network of specialists that serve as a reference at the national level for vascular malformations Yes No
28	Do you think that establishing a register for vascular anomalies in Austria could be useful? Yes No

The list of all the PCPs working in outpatient clinics was obtained from the Austrian Society of Pediatrics. Since a single source with all their contacts was not available, we created a mailing list of the PCPs including the 373 e-mail addresses that could be found in the web. The results are presented as the percentage of the response and the valid returned responses for a given question.

The survey was administered with LimeSurvey (LimeSurvey GmbH, Hamburg, Germany; URL http://www.limesurvey.org). Data were collected anonymously. The Fisheŕs exact test was used for group comparison in case of categorical data. Statistical significance was defined as *p* < 0.05. The study was approved by the ethics committee of the Medical University of Graz (32-236 ex 19/20).

## Results

Of the 373 PCPs contacted via email, one-fourth (*n* = 93, 25%) participated in the survey. 86 questionnaires were returned complete, while in 7 of them some questions were not answered. The response rate differed between the nine Austrian regions, with the highest response rate in Salzburg and the lowest in Charintia and Vorarlberg (Table 2).

**Table 2:** Overview of the Austrian states with each state's total population, population aged 18 years or less, number of primary care pediatricians that were sent our survey and response rate. Numbers were retrieved from Statistik Austria, kinder- und Jugendhilfestatistik für das Berichtsjahr 2021.

State	Population	Population <18 years	Number of invited PCP	Response rate *n* (%)
Burgenland	296,010	47,207	7	2 (28.6%)
Carinthia	562,089	90,558	26	2 (7.7%)
Lower Austria	1,690,879	296,058	61	18 (29.5%)
Upper Austria	1,495,608	271,925	49	11 (22.4%)
Salzburg	560,710	98,676	22	9 (40.9%)
Styria	1,247,077	201,675	64	24 (37.5%)
Tyrol	760,105	132,671	31	6 (19.4%)
Vorarlberg	399,237	76,655	24	4 (16.7%)
Vienna	1,920,949	330,860	89	17 (19.1%)
Austria	8,932,664	1,546,285	373	93 (24.9%)

PCP, primary care pediatrician.

Most outpatient clinics (72/93, 77%) were located less than 30 min by car from the next regional hospital with a reference pediatric department, and thus only 23% were in more isolated regions. The majority of the respondents reported to currently treat 1,000 or more pediatric patients aged 18 or less (54/93, 58%). Regarding their previous clinical experience relevant for VAs, 49/93 of the physicians (53%) reported experience in pediatric hemato-oncology, 38/93 (41%) in pediatric surgery, and 24/93 (26%) in pediatric dermatology.

### Infantile hemangiomas (IHs)

Almost half of the interviewed PCPs (39/91, 43%) stated to encounter between 15 and 30 patients with IH per year in their practice. The remaining PCPs visited either less than 15 (25/91, 28%) or more than 30 (27/91, 29%) patients with IH per year. More than half of the physicians (48/91, 53%) stated that they initiated therapy for less than 5% of the cases, about a third of the survey participants (26/91, 29%) further declared to start therapy with 5%–10% of their patients.

Most of the PCPs (85/90, 94%) declared to have some experience with the treatment options for IHs; 73/84 (87%) have worked with systemic beta-blockers, 41/84 (49%) with topical beta-blockers, 43/84 (51%) with LASER, 23/84 (27%) with surgery, and 10/84 (12%) with systemic corticosteroids. There was no significant association between previous work experience in relevant pediatric fields (*n* = 64 PCPs with and *n* = 27 PCPs without previous work experience in pediatric hemato-oncology, surgery or dermatology) and the proportion of patients with IHs seen in the practice per year (*p* = 0.957). Nevertheless, in case of a treatment indication, 76/89 (85%) of the interviewed PCPs referred the patient to a reference center. Location (86/87, 99%) and size (94%) of the IH were the most important parameters affecting the treatment choice according to the majority of PCPs, while 56/87 (64%) considered the number of lesions as important. About one third (33/86, 38%) considered appropriate to commence the treatment with beta-blockers the latest during the 4th month of life. 27/86 (31%) considered the 2nd month of life the minimum age for therapy initiation. The remaining physicians indicated the minimum age for treatment to be during or after the 6th month of life (26/86, 30%).

### Vascular malformations

More than two thirds of the physicians (58/86, 67%) have treated less than 5 patients with vascular malformations at the time of the survey, 24/86 (28%) took care of 5–10 patients and only 4/86 (5%) had more than 20 patients.

There was no significant association between previous work experience in relevant pediatric fields (*n* = 64 PCPs with and *n* = 27 PCPs without previous work experience in pediatric hemato-oncology, surgery or dermatology) and the number of patients treated for vascular malformations in the practice per year (*p* = 0.755).

Eighty-two of 86 (95%) PCPs reported that they did not use any compressive and anticoagulant therapy at the time of the survey. From our sample, 20/86 (23%) PCPs reported to have up to 5 patients who had undergone at least one surgical or radiological treatment for their vascular malformation.

### Reference network and educational opportunities

When asked about their possibility to refer the patients, the vast majority (78/86, 91%) declared to know a specialist or specialized center that they can turn to. Moreover, the location of the clinic (distance less or more than 30 min to the closest regional hospital) did not significantly influence the possibility of referring patients to a specialist in the field (less than 30 min: 53/65, 81.5% vs. more than 30 min 18/21, 85.7%; *p* > 0.99), an interdisciplinary group (less than 30 min: 30/65, 46.2% vs. more than 30 min 12/21, 57.1%; *p* = 0.455) or a reference center (less than 30 min: 57/68, 83.8% vs. more than 30 min 19/21, 90.5%; *p* = 0.725). Nevertheless, 72/86 (84%) declared not to know the ISSVA classification. Interestingly, 13/14 (93%) physicians who knew the classification also stated that the ISSVA classification is essential for the correct diagnosis and treatment of vascular anomalies.

Only 13/86 (15%) of the interviewed colleagues affirmed to know the national working group for vascular anomalies (Arbeitsgruppe für Interdisziplinäre Behandlung Vaskulärer Anomalien, AIVA) and even less (3/86, 4%) declared to know any dedicated Austrian patient association.

The increase of educational opportunities in the field of pediatric vascular anomalies was considered necessary by 53/86 PCPs (62%), while 38% considered the current situation sufficient.

### Future

There was broad consensus (84/86, 98%) about the importance of building a national network of specialists connected with the caregivers in the outpatient clinics. Furthermore, the majority of PCPs (79/86, 92%) agreed with the usefulness of establishing a registry of vascular anomalies in Austria.

## Discussion

The results of our survey highlight the insufficient awareness and networking in the field of vascular anomalies (VAs) among PCPs in Austria. Furthermore, the findings emphasize the need to endorse connections between patients, PCPs, and VA experts, with the aim of improved communication and specific education.

VAs account for a large and diverse group of mostly rare conditions. They can involve any type of vessel and appear anywhere in the body (9). The severity of their presentation varies from minor superficial capillary malformations without clinical implications to major vascular syndromes, causing functional impairments and affecting the quality of life of both patients and their families (10). The onset of clinical manifestations is possible at any age, from the perinatal period to adulthood. However, most of them are diagnosed during childhood (11).

In the recent medical literature the model of the “patient journey” has been developed describing the entire experience of a patient from hospital admission to discharge, aiming to improve the single steps of the process (12). In case of rare diseases, such as VAs, this “journey” covers the entire life of patients and their families, from the onset of symptoms to the demise (5)*.* Obtaining the correct diagnosis of VAs is the first essential step, but in case of orphan diseases, this process is challenging and requires experience in the field due to their rarity and broad spectrum of presentation (5). For this reason, the education of PCPs on VAs represents an essential prerequisite to reduce the delay between the onset of symptoms and the appropriate diagnosis (13).

We initially hypothesized that PCPs with previous experience in the fields of hemato-oncology, pediatric surgery, or pediatric dermatology would be more familiar with the management of VAs and would be connected with a network of specialists. However, we did not notice any difference based on training experiences. Recently published reports have shown that the distance of the outpatient clinic from specialized centers may be a factor possibly related to delayed diagnosis (14). According to our results, the presence of networking between PCPs and VA specialists did not vary according to the distance of PCPs from referral hospital.

The amount of information concerning IH available to physicians seems to be sufficient based on our survey's results. The majority of respondents provided answers reflecting the most recent diagnostic and therapeutic guidelines (15, 16). The increased degree of expertise in IH management is likely associated with the high incidence of this benign tumor. However, in the rare cases of high-risk IH, the fast recognition of features predisposing to complications, such as localization on the face or buttocks, size greater than 3 cm, and multifocality of lesions (>5) is essential (17, 18). This awareness has played an important role even during the Covid-19 pandemic, as PCPs have been able to provide patients with telemedicine consultations based on such parameters (18). The majority of our respondents described to have access to a consultation with an expert in case of red flags.

Our study also confirmed that the number of pediatric patients requiring treatment is relatively low, in accordance with the epidemiology of this kind of rare conditions. Therefore, PCPs are often not exposed to a sufficient number of cases to develop expertise in this field. Most of the PCPs who responded dealt with less than five patients with vascular malformations at the time of the survey. However, despite their rarity, patients with vascular malformations are chronically affected and require lifelong care associated with a significant socio-emotional and financial burden (19). This emphasizes the importance of centralized management of such pathologies in specialized centers and the necessity to improve and strengthen the network between PCPs and specialists (13).

In order to promote a uniform nomenclature for VAs worldwide, the ISSVA has drafted a classification of VAs that is updated every two years (20). The increasingly disseminated use of the correct taxonomy has simplified communication between caregivers and has served as a useful tool for diagnosing and treating VAs. Recent evidence has shown that incorrect or imprecise use of the ISSVA classification is related to inappropriate treatment (21). Unfortunately, only a minor part of our respondents was aware of this classification system, and interestingly, the PCPs who declared to use it considered it essential for the prompt recognition of complicated patients.

In 2011, a group of dedicated Austrian specialists started to meet on a yearly basis with the aim to provide certified education on VAs and a platform to discuss challenging cases on a national level. They have established the Austrian Society for the Multidisciplinary Management of Vascular Anomalies (Arbeitsgemeinschaft für Interdisziplinäre Behandlung Vaskulärer Anomalien, AIVA). Initiatives such as the AIVA promote the cooperation between professionals in different fields regarding VAs. For instance, pediatric surgeons, pediatricians, vascular surgeons, plastic surgeons, angiologists and other specialists, constitute the AIVA. Unfortunately, despite its intense activity over the years, only a small part of the participants was aware of the existence of this group. The participation of patients associations in the coordination of projects such as the AIVA offers an additional point of view and allows clinicians to get acquainted with peculiar difficulties experienced by each individual patient (22). In fact, patients may carry information not included in the expertise of healthcare professionals. Some authors state that this active interaction may improve the quality of life and even increase the survival rate of patients (23). Particularly for rare diseases like VAs, the connection between patients, caregivers and associations is essential. Our survey emphasized the imperative need of upgrading this cooperation in Austria.

The main limitation of the present study is that only one fourth of the addressed PCPs responded. A possible reason for that is the format of the survey, sent out as an email questionnaire. As stated by a recent study, physicians have a low propensity to respond to unpaid email-sent questionnaires with an average response rate of 18.2% (24). However, the level of participation in our work was homogenous among most Austrian states and thus it is reasonable to assume that the results reflect the real situation. A further limitation could also be the lack of experience highlighted by the PCP respondents from the survey especially concerning vascular malformations. This could also have led to a limited recognition of vascular malformations patients especially concerning vascular malformations, underestimating the real prevalence of these conditions.

In conclusion, this survey is the first attempt to understand the path of pediatric patients affected by VAs in Austria. It identifies the need of disseminating awareness of the established network of specialists and dedicated centers dealing with vascular anomalies, for example by creating a registry as well as an interactive national platform for discussing difficult cases. It also underlines the necessity to improve the educational opportunities and the connection with patients’ associations, for instance by spreading informative material on the activity of the expert centers. Furthermore, our results can be useful for further survey-based studies in other countries with similar healthcare settings.

## Data Availability

The raw data supporting the conclusions of this article will be made available by the authors, without undue reservation.
